# Characteristics of undiagnosed diabetes in men and women under the age of 50 years in the Indian subcontinent: the National Family Health Survey (NFHS-4)/Demographic Health Survey 2015–2016

**DOI:** 10.1136/bmjdrc-2019-000965

**Published:** 2020-02-24

**Authors:** Kajal T Claypool, Ming-Kei Chung, Andrew Deonarine, Edward W Gregg, Chirag J Patel

**Affiliations:** 1 Department of Biomedical Informatics, Harvard Medical School, Boston, Massachusetts, USA; 2 Human Health and Performance Systems, MIT Lincoln Laboratory, Lexington, Massachusetts, USA; 3 Department of Epidemiology and Biostatistics, Imperial College London, London, London, UK

**Keywords:** india, diagnosis, epidemiology, glucose

## Abstract

**Objective:**

Prior studies examining diabetes prevalence in India have found that nearly 50% of the diabetes population remains undiagnosed; however, the specific populations at risk are unclear.

**Research design and methods:**

First, we estimated the prevalence of undiagnosed diabetes in India for 750 924 persons between the ages of 15 years and 50 years who participated in the National Family Health Survey (NFHS-4)/Demographic Health Survey (2015–2016), a cross-sectional survey of all 29 states and 7 union territories of India. We defined ‘undiagnosed diabetes’ as individuals who did not know about their diabetes status but had high random (≥200 mg/dL) or fasting (≥126 mg/dL) blood glucose levels. Second, using Poisson regression, we associated 10 different factors, including the role of healthcare access, and undiagnosed diabetes. Third, we examined the association of undiagnosed diabetes with other potential comorbid conditions.

**Results:**

The crude prevalence of diabetes for women and men aged 15–50 years was 2.9%, 95% CI 2.9% to 3.1%, with self-reported diabetes prevalence at 1.7%, 95% CI 1.6 to 1.8. The overall prevalence of undiagnosed diabetes for 15–50 year olds was at 1.2%, 95% CI 1.2% to 1.3%. Forty-two per cent, 95% CI 40.7% to 43.4% of the individuals with high glucose levels were unaware of their diabetes status. Approximately 45%, 95% CI 42.9% to 46.4% of undiagnosed diabetes population had access to healthcare. Men, younger individuals, and those with lower levels of education were most at risk of being undiagnosed. Geographically, the Southern states in India had a significantly higher prevalence of undiagnosed diabetes despite having nearly universal access to healthcare. Risk factors combined with random glucose could predict undiagnosed diabetes (area under the curve of 97.8%, 95% CI 97.7% to 97.8%), Nagelkerke R^2^ of 66%).

**Conclusion:**

Close to half (42%) of the people with diabetes in India are not aware of their disease status, and a large subset of these people are at risk of poor detection, despite having health insurance and/or having access to healthcare. Younger age groups and men are the most vulnerable.

**Significance of this study**

**What is already known about this subject?**

- Evidence from prior studies indicates that diabetes in India is associated with higher socioeconomic status populations, predominantly in men and in the oldest individuals with high conversion rates from prediabetes to diabetes and from healthy to prediabetes (totaling nearly 45.1%).
- Evidence on the prevalence and risk factors for undiagnosed diabetes in India is limited to specific regions of the country, with the largest study covering 15 out of the 29 states of India. Given the geographic, ethnic and sociocultural diversity in India, it is difficult to draw any nationwide conclusions.

**What are the new findings?**

- Based on the largest, nationally representative survey, the fourth National Family Health Survey/Demographic Health Survey (NFHS-4/DHS) conducted in 2015–2016, of women aged 15–49 years and men aged 15–54 years and covering all 29 states and 7 union territories in India, our analysis provides risk factors associated with undiagnosed diabetes in India and, further, highlights the geographic discrepancies across the states of India. Our findings further draw attention to three aspects.
  - First, 42% of the individuals in India with diabetes are unaware of their diabetes status (are ‘undiagnosed’).
  - Second, there is poor detection of diabetes in India. Nearly 45% of undiagnosed diabetes individuals have access to healthcare.
  - Third, region of the country is a significant factor for undiagnosed diabetes more so than urban versus rural dwelling populations disproportionally effecting men and younger individuals.

**Significance of the study**

**How might these results change the focus of research or clinical practice?**

- Our findings suggest that access to healthcare should potentially be coupled with routine and rapid low-cost, serendipitous screening of individuals for high glucose levels. Further refinement of these results to the district level can aid in decision support for individual healthcare providers and tertiary healthcare centers throughout India to determine how and when to screen for diabetes.

## Introduction

Diabetes is the ninth leading cause of death in India.^1 2^ The International Diabetes Federation estimates the diabetes cases in India (in 2017) at nearly 73 million persons between the ages of 20 years and 79 years, a prevalence of nearly 10.4%.^3 4^ Half of this population might be unaware of their diabetes status,^3^ presenting a quandary for policy makers.^5^ The enormous size of the population of undiagnosed diabetes with higher proportions in the under 50 years of age means that non-identification of such cases before 50 years of age has the potential to seriously stress the healthcare system.

In this study, we identify characteristics of individuals with undiagnosed diabetes and analyze the dichotomy between poor awareness (undiagnosed diabetes) and poor detection of diabetes (those that remain undiagnosed despite access to healthcare) in participants of a cross-sectional survey called the fourth Indian National Family Health Survey (NFHS-4)/Demographic Health Surveys (DHS). The Indian Ministry of Health and Family Welfare conducted NFHS-4 between 2015 and 2016 on women of reproductive age (15–49 years) and their partners (15–54 years). The survey provides essential information on health and family welfare together with biometric measurements including height, weight, blood pressure, and blood glucose levels. We estimated the prevalences and identified the risk factors associated with poor awareness and poor detection of diabetes. Lastly, we examined the burden of undiagnosed diabetes on other comorbid conditions.

## Methods

### The NFHS and DHS 2015–2016

The NFHS-4/DHS conducted in 2015–2016 was designed to be nationally representative of the household population of women aged 15–49 years and men aged 15 –54 years covering all 29 states and 7 union territories in India.^6^ Participants were surveyed from 20 January 2015 to 4 December 2016. The survey used the 2011 Census of India as the sampling frame, with a two-stage sample stratification. The primary sampling units were villages in rural areas and the census enumeration blocks in urban areas and were selected with a probability proportional to the size within each stratum. All women aged 15–49 years who resided or spent the previous night in selected households were eligible for participation in the women’s survey. In a random subsample of about 15% of households, all men aged 15–54 years who resided or spent the night in these households were eligible for the men’s survey. In addition to survey questions, the survey included measurements of height, weight, blood pressure, and random blood glucose levels on participants. The survey response rate was nearly 98% at the household level and was 97% and 92% among eligible women and men, respectively. A total of 793 194 people (women: 684 845, men: 108 349) participated in the survey.

### Analytic sample

**Figure 1:**
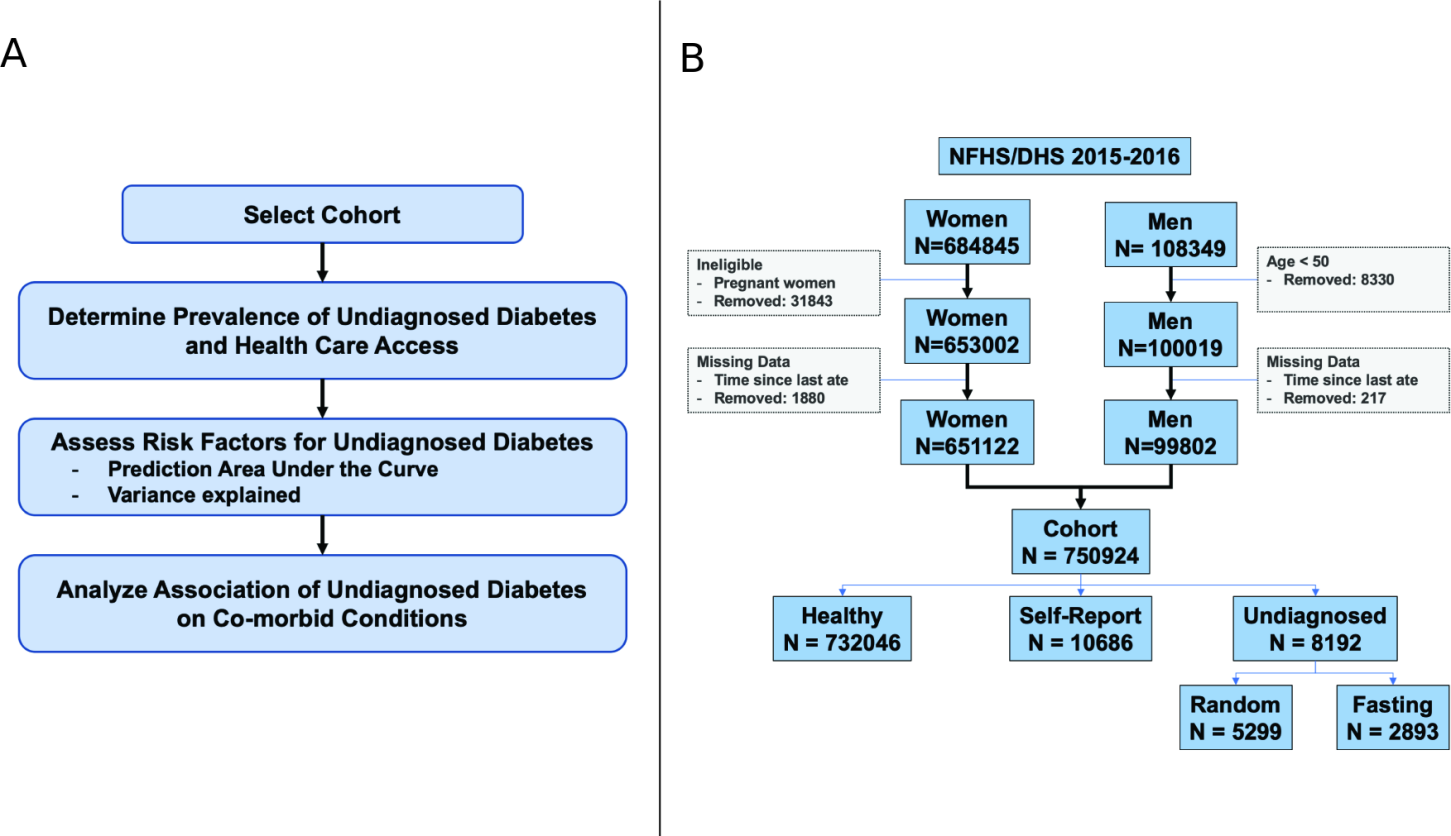
(A) Overall methodology; (B) cohort selection from the National Family Health Survey/Demographic Health Surveys (NFHS/DHS) conducted in India in 2015–2016.

We analyzed data on 750 924 participants from the 2015–2016 NFHS/DHS^6^ of India (see figure 1A for overall methodology). We analyzed both men and non-pregnant women under the age of 50 years separately and in combination (figure 1B).

### Diabetes definitions

Participants of the NFHS-4/DHS 2015–2016 had random blood glucose measured from a finger-stick blood specimen using the FreeStyle Optium H glucometer with glucose test strips. A referral form to a health facility for additional medical evaluation was provided for any respondent with random blood glucose ≥200 mg/dL. While individuals were not instructed to fast, the survey asked when participants last ate or drank.

The survey elicited information from all interviewed men and women on: (1) their health status including whether they currently have diabetes, asthma, heart disease, thyroid disorder or cancer; and (2) their access to healthcare to ascertain if the participant or a member of their household has health insurance, has seen a doctor in the past 12 months, and/or has visited a healthcare facility in the past 3 months.

We defined ‘self-report diabetes’ as all non-pregnant individuals who answered ‘yes’ to the question do you currently have diabetes. We defined ‘undiagnosed diabetes’ as participants who answered ‘no’ to the question do you currently have diabetes^7 8^ and following a laboratory assessment either had an opportunistic fasting glucose level ≥126 mg/dL (referred to as ‘fasting’) or had a random glucose level ≥200 mg/dL (referred to as ‘random’). We define ‘opportunistic fasting’ as individuals who self-reported that they had not eaten or had any calorie intake for 8 or more hours. This conforms to the US Preventive Services Task Force^9^ and the Research Society for the Study of Diabetes in India (RSSDI) guidelines (2017)^10^ for diabetes screening, where three tests can be used to screen for the presence of diabetes (including hemoglobin A1C (HbA1c), a fasting plasma glucose level ≥126 mg/dL, or oral glucose tolerance test glucose level of ≥200 mg/dL).

### Comorbid conditions

The NFHS-4/DHS survey also collected participants’ blood pressure measured using an Omron Blood Pressure Monitor to determine the prevalence of hypertension. Blood pressure measurements for each respondent were taken three times with an interval of 5 min between readings. Respondents whose average systolic blood pressure (SBP) was >140 mm Hg or average diastolic blood pressure (DBP) was >90 mm Hg were considered to have elevated blood pressure readings. We defined self-report hypertension as participants who answered ‘yes’ when asked if they were told by a doctor that they have high blood pressure or if they were currently taking any blood pressure medications.

All interviewed women and men in the NFHS-4/DHS survey were also asked whether they have asthma, thyroid disorder, heart disease or cancer. We defined self-reported heart disease and self-reported thyroid disorder as participants who answered ‘yes’ to these specific questions.

### Healthcare access

The NFHS-4/DHS survey participants were asked a series of questions to determine health insurance coverage, the sources of healthcare, and frequency of contact with healthcare workers/healthcare professionals. We defined healthcare access (yes/no) as either having health insurance, seeing a healthcare provider in the past 12 months, or visiting a healthcare facility in the past 3 months.

### Sociodemographic characteristics

We identified a set of potential sociodemographic risk factors of diabetes in India^11–14^ including sex, age, age groups (in 10-year bins), wealth index (poorest, poor, middle, rich, and richest), level of education (none, primary, secondary, and higher), body mass index (BMI) (kg/m^2^), smoking (in packs per day), drinking (in drinks per day), place of residence (urban vs rural) and state of residence (reference state: Gujarat). We grouped Indian states and union territories into the six administrative regions,^15^ including North, North East, Central, South, East, and West, to ensure adequate sample size within each region (reference region: Central).

The NFHS-4/DHS survey included a ‘wealth score’ based on the number and type of consumer goods in a household, such as television, bicycle or car, and housing characteristics such as source of drinking water, toilet facilities, and flooring materials^6^ and derived using principal component analysis. The survey compiled national wealth quintiles by assigning the household score to each usual (de jure) household member, ranking each person in the household population by their score, and then dividing the distribution into five equal categories, each with 20% of the population.^6^

### Statistical analysis

We computed prevalence and proportion estimates for diabetes, self-report and undiagnosed diabetes using sampling weights and survey-weighted proportions to account for the survey design. We expressed estimates as means with 95% CI. We derived prevalence ratios to examine the association of the sociodemographic exposures and outcomes (diabetes, self-report, undiagnosed) using a log-bionomial model implemented using survey-adjusted Poisson regression^16^ in R. We used this model to compute the prevalence ratio for each independent exposure using both univariate and fully adjusted models. We corrected all p values for multiple comparisons using the Bonferroni method and deemed all Bonferroni-adjusted p values<0.05 to be significant.

We assessed the accuracy with which individuals with undiagnosed diabetes can be predicted with sociodemographic variables (ie, sex, age, place of residence, region of residence, BMI, and lifestyle behaviors), in addition to comparable models for self reported diabetes. We trained two logistic regression models on a random sample of the three quarters of the entire cohort of undiagnosed and non-diabetic individuals. The first model included all sociodemographic variables described above, while the second model included (in addition) random glucose values. We report test accuracy and the area under the curve (AUC) for predicting undiagnosed diabetes in the left-out test cohort (the one quarter left-out sample).

Furthermore, we hypothesized that undiagnosed diabetes is associated with prevalence of self-reported comorbid conditions, namely hypertension, heart disease and thyroid disorders. To test this hypothesis, we used multivariate regression models to compute the difference in prevalences of comorbid conditions between persons with undiagnosed diabetes and those that self-reported.

## Results

**Figure 2:**
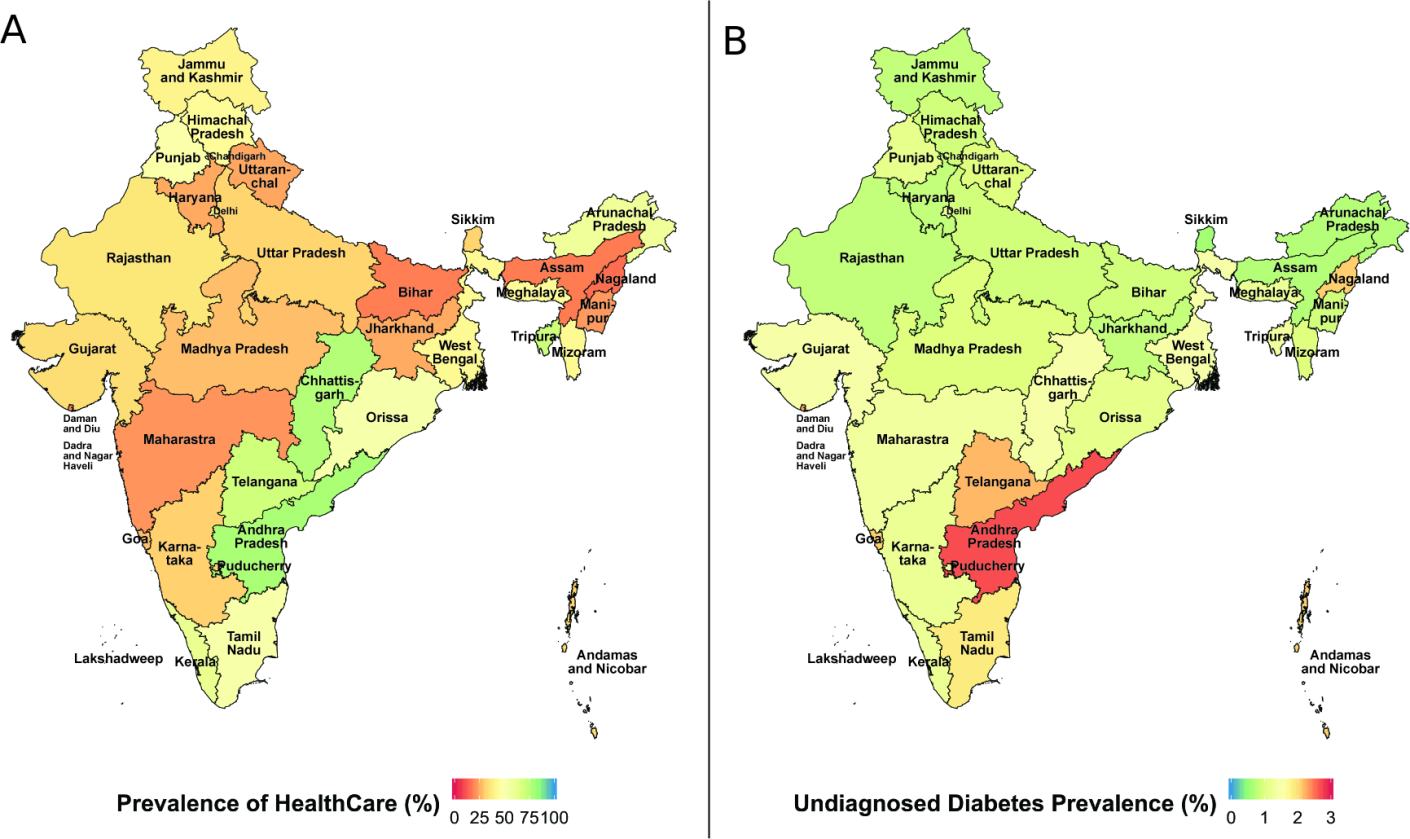
(A) Prevalence of healthcare access in India by state. (B) Prevalence of undiagnosed diabetes in India by state.

The NFHS4/India DHS in 2015–2016 surveyed a total of 753 038 non-pregnant individuals between the ages of 15 years and 50 years (online supplementary table S1). We removed 2114 individuals that were missing fasting or healthcare access status. This yielded a sample size of 750 924 (see figure 1B). Eighty-seven per cent of the surveyed individuals were women. Sixty-four per cent of the surveyed individuals were between 15 years and 35 years of age. Nearly two-thirds of the cohort population lived in rural areas with 63% belonging to the middle-class or higher. Thirty-eight per cent (45% for men and 37% for women) of this cohort had access to healthcare (online supplementary table S1). Figure 2A shows the prevalence of healthcare access for the different states.

10.1136/bmjdrc-2019-000965.supp1 Supplementary data

### Prevalence of undiagnosed diabetes

Table 1 provides the survey-adjusted prevalences for the self-report and undiagnosed diabetes groups. online supplementary table S2 provides the distribution of the diabetes population (self-report vs undiagnosed) stratified by sex. The crude prevalence of diabetes for men and women aged 15–50 years was estimated at 2.9%, 95% CI 2.9% to 3.1% with self-reported diabetes prevalence at 1.7%, (95% CI 1.6% to 1.8%) similar to the overall prevalence reported in reference.^6^ The overall prevalence of undiagnosed diabetes among 15–50 year olds was 1.2%, (95% CI 1.2% to 1.3%) (table 1). Forty-two per cent (95% CI 40.7% to 43.4%) of the individuals with diabetes were unaware (undiagnosed diabetes) of their diabetes status, and 27.6%, 95% CI 26.5% to 28.6% of the individuals with diabetes were undiagnosed with random glucose ≥200 mg/dL (table 1). A percentage of 50.5,(95% CI 47.2% to 53.7%) of men had undiagnosed diabetes versus 40.5%(95% CI 39.1% to 42.0%) women. Among those that were undiagnosed, 44.6%(95% CI 42.9% to 46.4%) had access to healthcare (men: 53%, 95% CI 48.9% to 57.1%, women: 42.7%, 95% CI 40.8% to 44.6%, online supplementary table S2).

**Table 1:** Survey-adjusted means and prevalence for self-report and undiagnosed diabetes groups

	Self reported diabetes	Undiagnosed diabetes		
		All	Healthcare	
			Yes	No
Unweighted sample size (diabetes n=18 878)	10 686	8192	3417 (8192)	4775 (8192)
Unadjusted prevalence (95% CI), % (overall: 2.9 (2.9 to 3.1))	1.7 (1.7 to 1.9)	1.2 (1.2 to 1.3)	N/A	N/A
Diabetes proportions (95% CI), %	58 (56.6 to 59.3)	42 (40.7 to 43.4)	44.6 (42.9 to 46.4)	55.4 (53.6 to 57.1)
Sex (95% CI), %				
Female	87 (85.8 to 88.2)	82 (80.6 to 83.4)	78 (75.8 to 80.2)	84 (82.3 to 85.7)
Male	13 (11.8 to 14.2)	18 (16.6 to 19.4)	22 (19.8 to 24.2)	16 (14.3 to 17.7)
Mean age (95% CI), years	39.1 (38.7,39.4)	37.8 (37.5 to 38.1)	38.26 (37.9 to 38.6)	37.41 (37.0 to 37.8)
Age categories (95% CI), %				
15–24	7 (6.1 to 7.9)	10 (9.1 to 10.9)	7 (5.8 to 8.2)	12 (10.7 to 13.3)
25–34	18 (16.8 to 19.3)	20 (18.7 to 21.3)	21 (19.0 to 23.0)	20 (18.3 to 21.7)
35–44	40 (38.6 to 41.5)	41 (39.4 to 42.6)	43 (40.6 to 45.4)	40 (37.8 to 42.2)
45–49	35 (33.4 to 36.6)	29 (27.5 to 30.5)	29 (26.8 to 31.2)	29 (27.0 to 31.0)
Wealth index (95% CI), %				
Poorest	8 (7 to 9)	10 (9.1 to 10.9)	9 (7.8 to 10.2)	11 (9.8 to 12.2)
Poor	11 (10.1 to 11.9)	15 (13.9 to 16.1)	14 (12.4 to 15.6)	16 (14.5 to 17.5)
Middle	16 (14.9 to 17.1)	21 (19.6 to 22.4)	22 (19.9 to 24.1)	21 (19.2 to 22.8)
Rich	28 (26.5 to 29.5)	27 (25.5 to 28.5)	29 (26.7 to 31.3)	25 (23.0 to 27.0)
Richest	36 (34.2 to 37.8)	27 (25.3 to 28.7)	26 (23.5 to 28.5)	27 (24.8 to 29.2)
Education (95% CI), %				
No education	24 (22.7 to 25.3)	31 (29.4 to 32.6)	31 (28.7 to 33.3)	31 (28.9 to 33.1)
Primary	15 (13.8 to 16.2)	15 (13.8 to 16.2)	16 (14.2 to 17.8)	14 (12.5 to 15.6)
Secondary	48 (46.5 to 49.5)	43 (41.4 to 44.7)	42 (39.6 to 44.5)	44 (41.8 to 46.2)
Higher	13 (11.8 to 14.2)	11 (9.8 to 12.2)	11 (9.2 to 12.8)	11 (9.5 to 12.5)
Place of residence (95% CI), %				
Urban	52 (49.7 to 54.4)	44 (41.9 to 46.1)	43 (40.1 to 45.9)	44 (41.4 to 46.6)
Rural	48 (45.7 to 50.4)	56 (53.9 to 58.1)	57 (54.1 to 59.9)	56 (53.4 to 58.6)
Region of country (95% CI), %				
Central	15 (13.9 to 16.1)	19 (17.8 to 20.2)	17 (15.4 to 18.6)	21 (19.5 to 22.6)
East	20 (18.2 to 21.8)	19 (17.3 to 20.7)	16 (13.9 to 18.1)	22 (19.7 to 24.3)
North	9 (8.2 to 9.8)	9 (8.2 to 9.8)	9 (7.9 to 10.1)	9 (8.0 to 10.0)
Northeast	2 (1.7 to 2.3)	2 (1.7 to 2.3)	2 (1.7 to 2.3)	3 (2.6 to 3.4)
South	42 (39.5 to 44.6)	34 (32.0 to 36.0)	47 (44.1 to 49.9)	24 (21.8 to 26.2)
West	12 (10.6 to 13.41)	16 (14.2 to 17.8)	10 (8.1 to 11.9)	21 (18.4 to 23.6)
Lifestyle				
Smokes (95% CI), %	13 (12.0 to 14.0)	15 (13.8 to 16.2)	19 (17.0 to 21.0)	12 (10.6 to 13.5)
Drinks (95% CI), %	7 (6.0 to 8.0)	8 (7.1 to 8.9)	11 (9.3 to 12.7)	5 (4.1 to 5.9)
Mean body mass index	25.4 (25.2 to 25.6)	25.3 (25.1 to 25.4)	25.6 (25.3 to 25.9)	25.0 (24.7 to 25.2)
Mean glucose (95% CI), mg/dL	172.4 (168.7 to 176.1)	234.0 (230.7 to 237.4)	231.5 (226.7 to 236.2)	236.1 (231.6 to 240.5)
Mean systolic BP (95% CI), mm Hg	123.5 (123.0 to 124.1)	125.4 (124.8 to 125.9)	125.5 (124.5 to 126.4)	125.3 (124.6 to 126.0)
Mean diastolic BP (95% CI), mm Hg	83.58 (83.1 to 84.1)	84.9 (84.3 to 85.5)	85.1 (84.3 to 85.9)	84.7 (83.8 to 85.6)
Access to healthcare (95% CI), %				
Yes	50 (48.1 to 51.9)	45 (43.3 to 46.7)	100 (0)	0 (0)
No	50 (48.1 to 51.9)	55 (53.26 to 56.74)	0 (0)	100 (0)

BP, blood pressure.

### Characteristics of undiagnosed diabetes population

Table 1 provides the survey-adjusted means for the self-report and undiagnosed diabetes group, highlighting the demographic, biological and lifestyle differences between the two groups. Compared with persons who self-reported diabetes, persons with undiagnosed diabetes were younger (39.1 years vs 37.8 years). Notably for persons aged 15–24 years, the prevalence of undiagnosed diabetes was 50% higher than the prevalence of self-reported diabetes for the same age group (self-reported: 7%, 95% CI 6.1% to 7.9%; undiagnosed: 10%, 95% CI 9.1% to 10.9%). A lower proportion of persons over the age of 45 years had undiagnosed diabetes (29%, 95% CI 27.5% to 30.5% compared with 35%, 95% CI 33.4% to 36.6% self-reported). Rural areas had a higher percentage of people with undiagnosed diabetes (rural: 56%, 95% CI 53.9% to 58.1% undiagnosed). In contrast, the percentage of people who reported as having diabetes in urban areas was higher (urban: 52%, 95% CI 49.7% to 54.4% self-report diabetes). Geographically, while the Southern and Eastern states had the highest proportions of diabetes, the Central and Western states of India had higher proportions of undiagnosed diabetes when compared with the self-report proportions. Higher proportions of people belonging to poorest, poor or middle classes (46% undiagnosed compared with 35% self-report) were identified as having undiagnosed diabetes with lower prevalence in undiagnosed diabetes for the upper two classes (table 1).

As expected, individuals with undiagnosed diabetes had higher blood glucose levels (undiagnosed: 234.0 mg/dL, 95% CI 230.7 to 237.4 mg/dL; self-report: 172.4 mg/dL, 95% CI 168.7 to 176.1mg/dL). The undiagnosed diabetes group also had higher blood pressure levels (undiagnosed systolic: 125.4 mm Hg, 95% CI 124.8 to 125.9 mm Hg; self-report systolic: 123.5 mm Hg, 95% CI 123.0 to 124.1 mm Hg; undiagnosed diastolic: 84.9 mm Hg, 95% CI 84.3 to 85.5 mm Hg; self-report: 83.6 mm Hg, 95% CI 83.1 to 84.1 mm Hg). This difference in blood pressure was not clinically significant (both groups were in the elevated blood pressure category as per the American College of Cardiology/American Heart Association (ACC/AHA) guidelines^17^).

### Differences in the undiagnosed diabetes population

Online supplementary table 3 highlights the differences within the undiagnosed diabetes populations, that is, between those that were undiagnosed with a non-fasting random glucose level ≥200 mg/dL and those that had a fasting random glucose level ≥126 mg/dL. A high percentage, 65.5 of undiagnosed diabetes population, had random glucose levels ≥200 mg/dL compared with the fasting group (random glucose level ≥126 mg/dL and time since they last ate ≥8 hours). Participants with random undiagnosed diabetes were older (39.37 years vs 34.79 years) and had on average higher BMI (26.2 kg/m^2^ vs 23.5 kg/m^2^) compared with persons with fasting undiagnosed diabetes. A higher percentage of persons with random undiagnosed diabetes lived in urban areas (46% compared with 40%) with some key regional/state level differences. We observed the biggest differences in the Western region where 13%, 95% CI 11.3% to 14.7% had random undiagnosed diabetes compared with 22% (95% CI 18.7% to 25.3%) with fasting undiagnosed diabetes (see online supplementary table S3). There was no significant difference in access to healthcare between the random and the fasting undiagnosed groups.

### Sociodemographic associations with undiagnosed diabetes versus self-reported diabetes

Table 2 shows the mean differences in the prevalence of undiagnosed diabetes when compared with self-report diabetes for the sociodemographic, biological (including comorbid conditions), and lifestyle factors using a fully adjusted model. online supplementary table S4 summarizes the mean differences obtained from univariate models. The undiagnosed population was different than those who reported diabetes. Undiagnosed diabetes (vs self-report diabetes) was associated with sex, age, education, state of residence, BMI, and lifestyle behaviors (such as smoking and drinking) (table 2, see online supplementary table S4 for univariate models). Women had a 28% lower prevalence of undiagnosed diabetes than men (Prevalence Ratio (PR)=0.72, 95% CI 0.67 to 0.79, p<0.0001). Older age groups (45–49 year olds) had a 26% lower prevalence of undiagnosed diabetes (PR=0.74, 95% CI 0.66 to 0.82, p<0.0001) than 15–24 year olds. Individuals aged 24–34 years who had access to healthcare had a 28% higher prevalence of undiagnosed diabetes (PR=1.28, 95% CI 1.11 to 1.49, p<0.05) than 15–24 year olds. Persons with higher education had a 17%–20% lower prevalence of being undiagnosed compared with those with no education. Overall, higher BMI was associated with increased prevalence of both diabetes and undiagnosed diabetes. A 1-unit increase in BMI was associated with a 1% increase in prevalence of undiagnosed diabetes (PR=1.01, 95% CI 1.01 to 1.02, p<0.001). Persons in the Eastern, Northern and Southern regions had lower prevalence of undiagnosed diabetes compared with the Central region. However, in the Southern states, persons with access to healthcare had a nearly 54% higher prevalence of undiagnosed diabetes (PR=1.54, 95% CI 1.41 to 1.67, p<0.0001). Last, persons who smoked and had access to healthcare had a 37% higher prevalence of undiagnosed diabetes (PR=1.37, 95% CI 1.24 to 1.52, p<0.0001).

**Table 2:** Comparing adjusted prevalence of undiagnosed diabetes with self-report diabetes and the mean difference for prevalence of comorbid conditions

	Diabetes versus healthy Prevalence ratio (95% CI)	Undiagnosed versus selfreport Prevalence ratio (95% CI)	Undiagnosed with healthcare versus undiagnosed without healthcare Prevalence ratio (95% CI)
Sex (ref: male)			
Female	0.95 (0.87 to 1.04)	0.72(0.67to0.79)***	0.95 (0.84 to 1.06)
Age category (ref: 15–24 years)			
25–34	2.09(1.91to2.29)***	0.9 (0.83 to 0.99)	**1.28(1.11to1.49).**
35–44	4.89(4.46to5.37)***	0.83(0.76to0.91)*	**1.26(1.09to1.45).**
45–49	8.5(7.66to9.42)***	0.74(0.66to0.82)***	1.19 (1.02 to 1.38)
Wealth (ref: poorest)			
Poor	1.12 (1.03 to 1.23)	1.1 (1 to 1.21)	1.03 (0.9 to 1.18)
Middle	1.31(1.2to1.44)***	1.14 (1.03 to 1.25)	1.13 (0.98 to 1.3)
Rich	1.67(1.52to1.84)***	0.99 (0.89 to 1.09)	1.15 (1 to 1.32)
Richest	1.89(1.7to2.09)***	0.85 (0.76 to 0.96)	1.16 (0.99 to 1.36)
Education level (ref: no education)			
Primary	1.17(1.1to1.26)**	0.84(0.78to0.91)**	1.01 (0.91 to 1.12)
Secondary	**1.06(1to1.13)**	0.8(0.75to0.85)***	0.95 (0.87 to 1.05)
Higher	0.92 (0.83 to 1.02)	**0.83(0.74to0.92).**	1.02 (0.88 to 1.19)
Body mass index (kg/m^2^)	1.06(1.05to1.06)***	1.01(1.01to 1.02)**	1 (1 to 1.01)
Residence (ref: urban)			
Rural	0.84(0.8to0.9)***	1.08 (1.01 to 1.15)	1.09 (1 to 1.18)
Region (ref: central)			
East	1.43(1.32to1.54)***	0.83(0.76to0.9)**	0.92 (0.81 to 1.04)
North	0.83(0.77to0.89)***	**0.89(0.82to0.96).**	1.08 (0.96 to 1.2)
Northeast	0.98 (0.9 to 1.07)	0.87 (0.79 to 0.96)	0.69(0.59to0.81)**
South	1.66(1.55to1.79)***	0.8(0.74to0.87)***	1.54(1.41to1.67)***
West	1.01 (0.93 to 1.1)	1.07 (0.98 to 1.16)	0.7(0.59to0.84)*
Smokes (ref: no smoking)	1.04 (0.96 to 1.12)	0.94 (0.87 to 1.02)	1.37(1.24to 1.52)***
Drinks (ref: no drinking)	1.15 (1.02 to 1.29)	0.94 (0.84 to 1.05)	1.07 (0.94 to 1.2)
Healthcare	1.13(1.08to1.19)***	0.92 (0.87 to 0.98)	NA
Mean diff (95% CI)	Mean diff (95% CI)	Mean diff (95% CI)
Glucose (95% CI), mg/dL	91.5(88.7to94.3)***	66.2(6.5to70.8)***	−11.4 (−17.6 to −5.2)
Systolic BP (95% CI), mm Hg	4.2(3.9to4.6)***	2.3(1.6to3.1)***	−0.5 (−1.6 to 0.6)
Diastolic BP (95% CI), mm Hg	2.6(2.2to3.0)***	1.4 (0.6 to 2.2)	−0.37 (−1.4 to 0.7)
Self-report:		
Hypertension (95% CI), %	7.5(6.5to8.4)***	−4.1(−5.9to−2.3)*	1.1 (1.3)
Heart disease (95% CI), %	4.5(3.8to5.2)***	−9.0(−10.3to−7.7)***	1.4 (0.5 to 2.3)
Thyroid disorder (95% CI), %	4.3(3.5to5.1)***	−7.6(−9.1to−6.1)***	3.1 (1.5 to 4.6)

Univariate prevalence ratios and adjusted prevalence ratios for men and women are given in online supplementary tables 4-6. Bonferroni-corrected p values are denoted as follows: corrected p value <0.0001 (***), corrected p value <0.001 (**), corrected p value <0.01 (*), corrected p value <0.05 (.). Factors that are associated with increased prevalence of undiagnosed (diabetes, undiagnosed with healthcare access) are shown in red. Factors associated with decreased prevalence of undiagnosed (diabetes, undiagnosed with healthcare access) are shown in blue. Factors that are not significant are given in black.

BP, blood pressure.

### Variance explained of undiagnosed diabetes in the overall population

Overall, our analyzed factors explained nearly 9% of the variance (R^2^=0.09) for undiagnosed diabetes versus individuals without diabetes (excluding the self-report diabetes group) and nearly 66% (R^2^=0.66) of the variance when combined with random glucose measures. These factors had an AUC of 74.8% (AUC=74.8%, 95% CI 74.7% to 74.9%) and an accuracy of 98.9% (95% CI 98.8% to 98.9%) when discriminating individuals with undiagnosed diabetes from individuals with no diabetes. When combining these factors with random glucose test values, these factors had an AUC of 97.8% (95% CI 97.7% to 97.8%) and an accuracy of 99.5% (95% CI 99.53% to 99.57%). When discriminating the undiagnosed diabetes population from the self-report diabetes group, these factors explained 6% of the variance (R^2^=0.06) and nearly 19% when combined with random glucose measures. The model had an area under the curve (AUC) of 60.4% (95% CI 59.4% to 61.3%) and an accuracy of 58.3% (95% CI 57.6% to 58.9) when classifying undiagnosed diabetes. With random glucose values, the AUC for discriminating undiagnosed diabetes from self-report individuals is 73.2% (95% CI 72.3% to 74%) with an accuracy of 66.3% (95% CI 65.6% to 67.0%). Online supplementary table S2 gives the comparative AUCs for these models.

### Undiagnosed diabetes associated with lower prevalence of heart disease

Table 2 highlights the differences in the glucose, SBP and DBP levels, and the prevalence of comorbid conditions between the undiagnosed and self-report diabetes groups. In online supplementary table S6, we highlight the differences of the same between women and men and examine the differences with respect to healthcare access. Glucose levels between the undiagnosed, and the self-report groups was significantly different (66.2 mg/dL, 95% CI 61.5 to 70.8 mg/dL, p<0.0001), after adjusting for sociodemographic, geographic and lifestyle factors. Individuals with undiagnosed diabetes also on average had slightly higher systolic (2.3 mm Hg, 95% CI 1.6 to 3.1 mm Hg, p<0.00001) and diastolic (1.4 mm Hg, 95% CI 0.6 to 2.2 mm Hg, p<0.1) blood pressure when compared with the self-report diabetes group (table 2).

As shown in table 2, heart disease prevalence in the undiagnosed group was 9.0% (95% CI −10.3% to –7.7%, p<0.00001) lower than the prevalence in the self report group. This difference was significantly pronounced in men with a difference of 13.7% (95% CI −18.1% to 9.2%, p<0.00001) (online supplementary table S6). Hypertension and thyroid disorder prevalence were both significantly lower in the undiagnosed group (hypertension: −4.1%, 95% CI −5.9%, to 2.3%, p<0.005, thyroid disorder: −7.6%, 95% CI −9.1% to 6.1%, p<0.00001, see table 2).

Among persons with undiagnosed diabetes, prevalence of comorbid conditions were not associated with access to healthcare (see online supplementary table S6).

## Discussion

A significant portion of the diabetes population in India, at least 42%, remains unaware of their diabetes status, and an overwhelming subset of this population (approximately 45%) is at risk of poor detection: undiagnosed diabetes despite having access to healthcare. This finding highlighting the poor awareness (undiagnosed) and poor detection of diabetes (undiagnosed with access to healthcare) in India is troubling from several aspects.

In addition to the high proportion of undiagnosed cases, our study had four key findings. First, men are more likely to be unaware of their diabetes status and more vulnerable to poor detection of diabetes compared with women. Our findings on poor diabetes detection in men conforms to the overall trends in diabetes—lower crude prevalence of diabetes in women (7.3%, 95% CI 7.1% to 7.4%) compared with 7.8%, 95% CI 7.6% to 8.0% in men^11^) and a significantly higher prevalence of diabetes (10% higher prevalence of diabetes relative to women; online supplementary table S2)—reported here and in prior studies.^11 14 18^ Furthermore, men had a nearly 10% higher prevalence of being undiagnosed despite having healthcare access compared with women in the same category.

Second, younger age groups are more likely to be unaware of their diabetes status and susceptible to poor diabetes detection. The proportion of 15–39 year olds with undiagnosed diabetes (both with and without access to healthcare) was nearly double the proportion of individuals that reported having diabetes for the same age categories. Overall, a 10-year increase in age lowered the prevalence of poor awareness by 10%. These findings are of particular concern given the additional burden that this population is likely to place on an already strained healthcare system. These findings also highlight the need to perhaps revisit the recommended age for routine screening of diabetes: the American Diabetes Association recommends routine diabetes screening for overweight and obese individuals of age ≥40 years and for others at age ≥45 years.^19^

Third, perhaps not surprisingly, individuals with higher education levels are more likely to be aware of their diabetes status. Individuals with higher levels of education (from primary to higher secondary) had a nearly 20% lower prevalence of undiagnosed diabetes when compared with individuals with no education. Thus, while it is reassuring to see that higher education reduces the prevalence of poor awareness of diabetes, it highlights the disparity in health outcomes associated with unequal access to education in India. Socioeconomic status and education did not significantly alter the prevalence of poor detection.

Lastly, the Eastern, North-Eastern and Southern regions of India all showed higher levels of diabetes awareness when compared with the Central states. Fewer individuals in these states were undiagnosed compared with the Central states. Despite having the highest access to healthcare (55.9%) and health insurance (45.4%), the Southern region in India had a significantly higher prevalence of poor detection compared to the Central region. Individuals in the Southern region had a nearly 54% higher prevalence of poor detection (PR=1.5, 95% CI 1.4% to 1.7%, p<0.0001) when compared with the Central region.

We also found that poor awareness of diabetes is associated with lower prevalence of comorbid conditions in India (vs self-report diabetes). We claim that this could be attributed to the younger age of the cohort or potential under-reporting of comorbid conditions. Given that nearly 45% of these undiagnosed individuals have healthcare access, we posit that providing healthcare access alone to individuals may not be sufficient and/or should be coupled with screening using random glucose tests.^20–25^ This is also in accordance with the RSSDI guidelines that specify that screening should be implemented ‘based on the prevalence of undiagnosed diabetes and available support from healthcare’.^10^

Our study has several strengths. While several studies have reported on the undiagnosed and the prediabetes population in India,^14 18^ this population has not been examined in the context of access to healthcare. We are, to the best of our knowledge, the first to examine the undiagnosed diabetes population in India with and without access to care. We emphasize that our analysis is representative of all 29 states and 7 union territories in India and includes both urban and rural regions, in contrast to studies that have focused on individual states and cities^5 26–28^ or subsets of states and union territories.^11 14^

However, our study also has several limitations. First, our estimates of undiagnosed diabetes are based on random glucose (capillary blood glucose (CBG)) measurements and opportunistic fasting information. While we used self-report information on an individual’s calorie intake to attain opportunistic fasting information, it does not meet the standards of diabetes diagnosis that call for using fasting venous plasma glucose, repeat measurements, or HbA1c.^29^ Since our random glucose definition is conservative (specific, but less sensitive), our estimates of undiagnosed diabetes are likely an underestimate, and the degree of underestimation is likely to be greater in younger people because they were over-represented among those having random measurements.^22 30–33^ Second, our sample of persons with undiagnosed diabetes was skewed towards persons with random glucose measurements ≥200 mg/dL (nearly 65% of the undiagnosed population), potentially biasing the assessment of the prevalences. Third, our analysis and findings are limited to 15–49 year old non-pregnant women and men. Our results do not include children ≤14 years of age or individuals ≥50 years. Furthermore, given that we only had access to random blood glucose readings, we are unable to make any distinction between type 1 and type 2 diabetes. Finally, the dataset was heavily skewed towards females, which could result in greater misrepresentation of the problem in men compared with women.

In conclusion, while prior studies have reported undiagnosed diabetes as high as 47% of the overall diabetes population^14^ for a subset of the Indian states, we extend these findings to provide a representation of the undiagnosed population across India and for a younger age demographic (15–49 years in women and men). We are, to the best of our knowledge, also the first to highlight that for certain age demographics (the younger age groups) and regions of the country (eg, in the Southern states of India) a high proportion of the diabetes population remains undiagnosed despite access to healthcare. These findings are especially of great importance as India works to put national attention on non-communicable diseases through its National Programme for Prevention and Control of Cancer, Diabetes, Cardiovascular Diseases and Stroke established in 2010 and its focus on bringing healthcare access to the poorest in the nation through the recent establishment of the Ayushman Bharat, the National Protection Mission.

## References

[1] India: Health Metrics [Internet]. Institute for health metrics and evaluation., 2015 Available: http://www.healthdata.org/india [Accessed cited 2019 Jul 14].

[2] The Lancet Lancet Global Burden of Disease [Internet], 2019 Available: https://www.thelancet.com/gbd [Accessed 2019 Jul 14].

[3] Cho NH, Shaw JE, Karuranga S, et al IDF diabetes atlas: global estimates of diabetes prevalence for 2017 and projections for 2045. Diabetes Res Clin Pract 2018;138:271–81. 10.1016/j.diabres.2018.02.023 29496507

[4] IDF IDF diabetes atlas - Home [Internet], 2019 Available: https://diabetesatlas.org/ [Accessed 2019 Apr 26].

[5] Tripathy JP, Thakur JS, Jeet G, et al Prevalence and risk factors of diabetes in a large community-based study in North India: results from a steps survey in Punjab, India. Diabetol Metab Syndr 2017;9:8 10.1186/s13098-017-0207-3 28127405 PMC5259959

[6] India National Family Health Survey (NFHS-4) 2015-16 [FR339], 2016 Available: https://dhsprogram.com/pubs/pdf/FR339/FR339.pdf

[7] Corsi DJ, Subramanian SV Socioeconomic gradients and distribution of diabetes, hypertension, and obesity in India. JAMA Netw Open 2019;2:e190411 10.1001/jamanetworkopen.2019.0411 30951154 PMC6450330

[8] Alberti KG, Zimmet PZ, Definition ZPZ Definition, diagnosis and classification of diabetes mellitus and its complications. Part 1: diagnosis and classification of diabetes mellitus provisional report of a who consultation. Diabet Med 1998;15:539–53. 10.1002/(SICI)1096-9136(199807)15:7<539::AID-DIA668>3.0.CO;2-S 9686693

[9] Siu AL, U S Preventive Services Task Force Screening for abnormal blood glucose and type 2 diabetes mellitus: U.S. preventive services Task force recommendation statement. Ann Intern Med 2015;163:861–8. 10.7326/M15-2345 26501513

[10] Bajaj S RSSDI clinical practice recommendations for the management of type 2 diabetes mellitus 2017. Int J Diabetes Dev Ctries 2018;38:1–115. 10.1007/s13410-018-0604-7 29527102 PMC5838201

[11] Geldsetzer P, Manne-Goehler J, Theilmann M, et al Diabetes and hypertension in India: a nationally representative study of 1.3 million adults. JAMA Intern Med 2018;178:363–72. 29379964 10.1001/jamainternmed.2017.8094 PMC5885928

[12] Agrawal S, Ebrahim S Prevalence and risk factors for self-reported diabetes among adult men and women in India: findings from a national cross-sectional survey. Public Health Nutr 2012;15:1065–77. 10.1017/S1368980011002813 22050916 PMC3458429

[13] Wells JCK, Pomeroy E, Walimbe SR, et al The elevated susceptibility to diabetes in India: an evolutionary perspective. Front Public Health 2016;4:145 10.3389/fpubh.2016.00145 27458578 PMC4935697

[14] Anjana RM, Deepa M, Pradeepa R, et al Prevalence of diabetes and prediabetes in 15 states of India: results from the ICMR-INDIAB population-based cross-sectional study. Lancet Diabetes Endocrinol 2017;5:585–96. 10.1016/S2213-8587(17)30174-2 28601585

[15] Wikipedia contributors Administrative divisions of India [Internet]. Wikipedia, The Free Encyclopedia 2019.

[16] Greenland S Model-Based estimation of relative risks and other epidemiologic measures in studies of common outcomes and in case-control studies. Am J Epidemiol 2004;160:301–5. 10.1093/aje/kwh221 15286014

[17] Whelton PK, Carey RM, Aronow WS, et al 2017 ACC/AHA/AAPA/ABC/ACPM/AGS/APhA/ASH/ASPC/NMA/PCNA guideline for the prevention, detection, evaluation, and management of high blood pressure in adults: a report of the American College of Cardiology/American heart association Task force on clinical practice guidelines.. Hypertension 2018;71:e13––115. 29133356 10.1161/HYP.0000000000000065

[18] Gupta A, Gupta R, Sarna M, et al Prevalence of diabetes, impaired fasting glucose and insulin resistance syndrome in an urban Indian population. Diabetes Res Clin Pract 2003;61:69–76. 10.1016/S0168-8227(03)00085-8 12849925

[19] American Diabetes Association Screening for diabetes. Diabetes Care 2002;25:S21–4. 10.2337/diacare.25.2007.S21 12502615

[20] Bragg F, Li L, Bennett D, et al Association of random plasma glucose levels with the risk for cardiovascular disease among Chinese adults without known diabetes. JAMA Cardiol 2016;1:813–23. 10.1001/jamacardio.2016.1702 27437922

[21] Moebus S, Göres L, Lösch C, et al Impact of time since last caloric intake on blood glucose levels. Eur J Epidemiol 2011;26:719–28. 10.1007/s10654-011-9608-z 21822717 PMC3186886

[22] Ziemer DC, Kolm P, Foster JK, et al Random plasma glucose in serendipitous screening for glucose intolerance: screening for impaired glucose tolerance study 2. J Gen Intern Med 2008;23:528–35. 10.1007/s11606-008-0524-1 18335280 PMC2324161

[23] Bowen ME, Xuan L, Lingvay I, et al Performance of a random glucose case-finding strategy to detect undiagnosed diabetes. Am J Prev Med 2017;52:710–6. 10.1016/j.amepre.2017.01.023 28279547 PMC5438773

[24] Friedman SM, Vallipuram J, Baswick B Incidental findings of elevated random plasma glucose in the ED as a prompt for outpatient diabetes screening: a retrospective study. BMJ Open 2013;3:e003486 10.1136/bmjopen-2013-003486 PMC3884805 24353254

[25] Bowen ME, Xuan L, Lingvay I, et al Random blood glucose: a robust risk factor for type 2 diabetes. The Journal of Clinical Endocrinology & Metabolism 2015;100:1503–10. 10.1210/jc.2014-4116 25650899 PMC4399288

[26] Anjana RM, Shanthi Rani CS, Deepa M, et al Incidence of diabetes and prediabetes and predictors of progression among Asian Indians: 10-year follow-up of the Chennai urban rural epidemiology study (cures). Diabetes Care 2015;38:1441–8. 10.2337/dc14-2814 25906786

[27] Kutty VR, Soman CR, Joseph A, et al Type 2 diabetes in southern Kerala: variation in prevalence among geographic divisions within a region. Natl Med J India 2000;13:287–92. 11209482

[28] Hamid Zargar A, Kariem Khan A, Rashid Masoodi S, et al Prevalence of type 2 diabetes mellitus and impaired glucose tolerance in the Kashmir Valley of the Indian subcontinent. Diabetes Res Clin Pract 2000;47:135–46. 10.1016/S0168-8227(99)00110-2 10670914

[29] American Diabetes Association 2. Classification and Diagnosis of Diabetes: Standards of Medical Care in Diabetes—2018. Diabetes Care 2018;41:S13–27. 10.2337/dc18-S002 29222373

[30] Narayan KMV, Chan J, Mohan V Early identification of type 2 diabetes: policy should be aligned with health systems strengthening. Diabetes Care 2011;34:244–6. 10.2337/dc10-1952 21193623 PMC3005469

[31] Andersson DKG, Lundblad E, Svärdsudd K A model for early diagnosis of type 2 diabetes mellitus in primary health care. Diabet Med 1993;10:167–73. 10.1111/j.1464-5491.1993.tb00036.x 8458195

[32] Engelgau MM, Thompson TJ, Smith PJ, et al Screening for diabetes mellitus in adults: the utility of random capillary blood glucose measurements. Diabetes Care 1995;18:463–6. 10.2337/diacare.18.4.463 7497854

[33] Rolka DB, Narayan KMV, Thompson TJ, et al Performance of recommended screening tests for undiagnosed diabetes and dysglycemia. Diabetes Care 2001;24:1899–903. 10.2337/diacare.24.11.1899 11679454

