# Supplementary material for: Characteristics of undiagnosed diabetes in men and women under the age of 50 years in the Indian subcontinent: the National Family Health Survey (NFHS-4)/Demographic Health Survey 2015–2016
Source: BMJ Open Diabetes Res Care. 2020 Feb 24;8(1):e000965. doi: 10.1136/bmjdrc-2019-000965 (PMC7206915; doi:10.1136/bmjdrc-2019-000965)
Supplement: Supplementary data [file bmjdrc-2019-000965supp001.pdf]

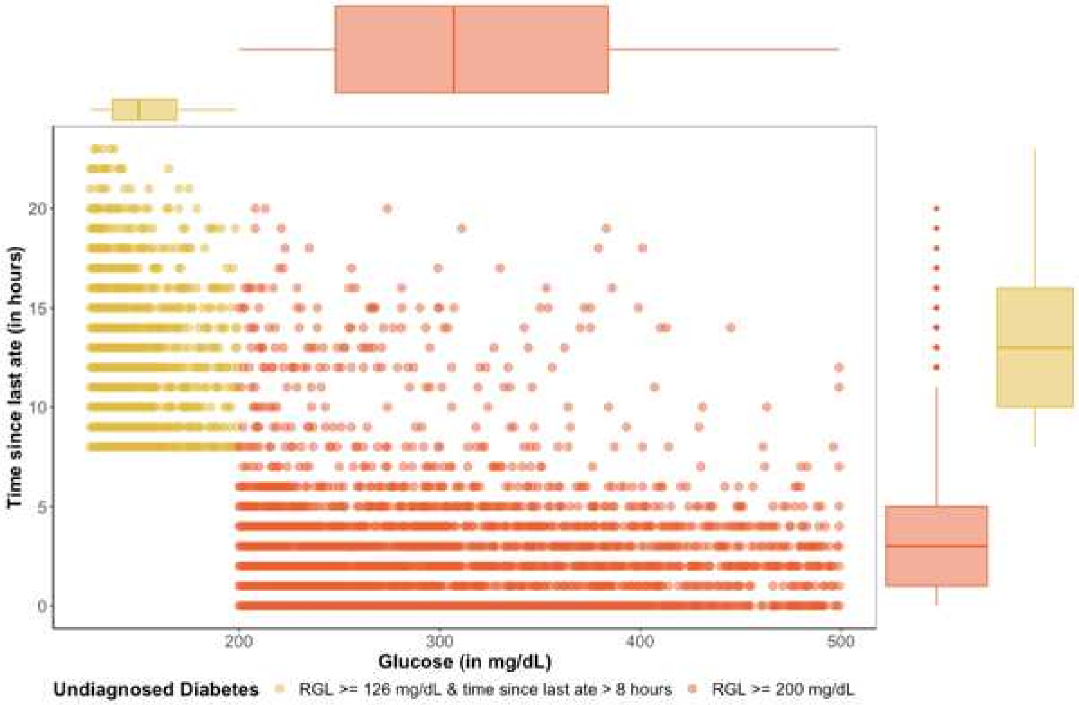

Figure S1. Distribution of random glucose levels in persons with undiagnosed diabetes shown as a function of the time since they last ate.

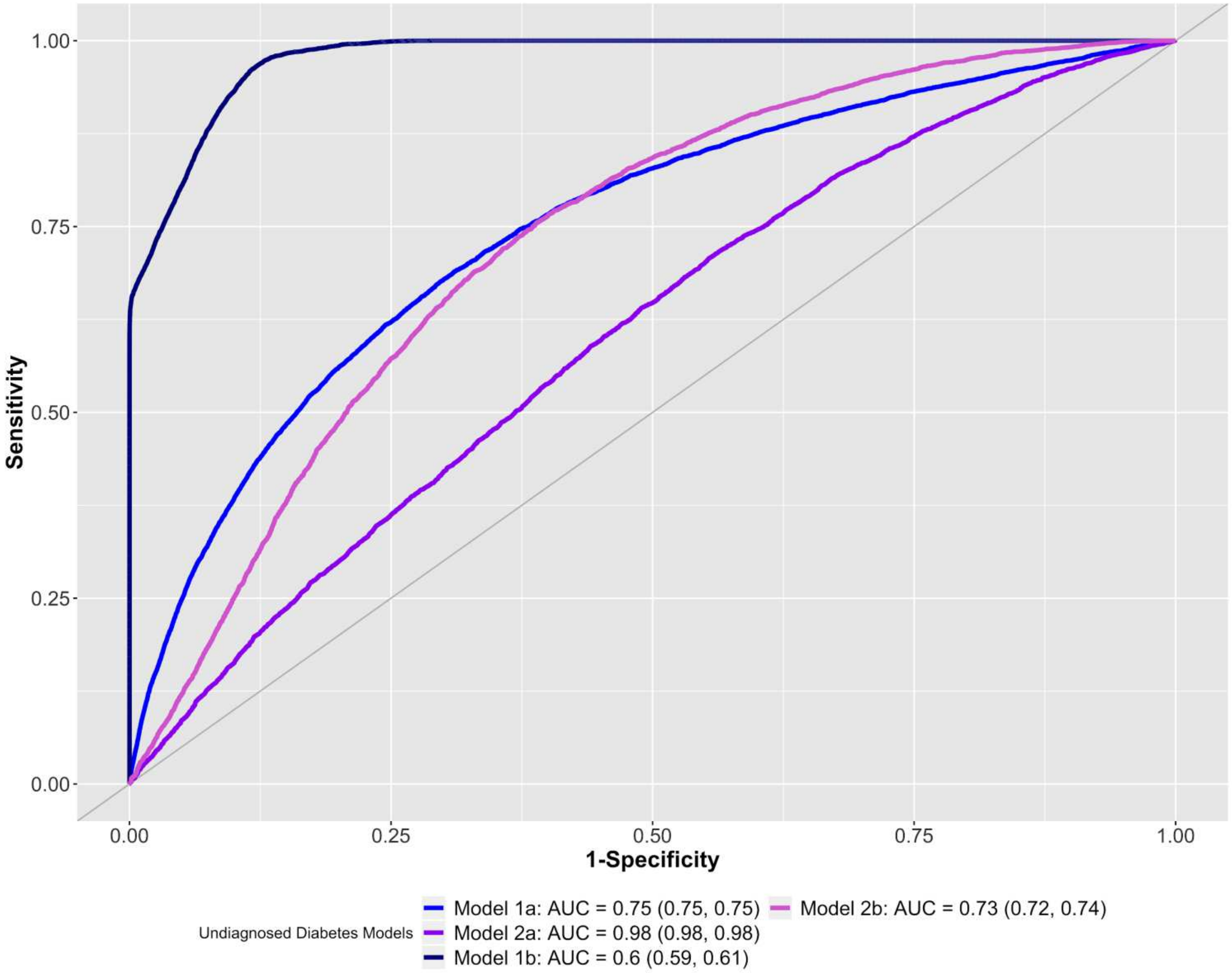

Figure S2. Receiver Operating Characteristic (ROC) Curves for predicting undiagnosed diabetes cases based on the factors in Table 2. The x-axis represents the specificity while the y-axis represents the sensitivity of the models. The lines shows the ROC curves for four different models. Model 1a classifies the undiagnosed diabetes from all individuals that don't have diabetes using the factors in Table 2. Model 1b discriminates the undiagnosed diabetes from the self-report diabetes using the factors in Table 2. Models 2a and 2b perform the classifications as specified for 1a and 1b and now including the random glucose values.

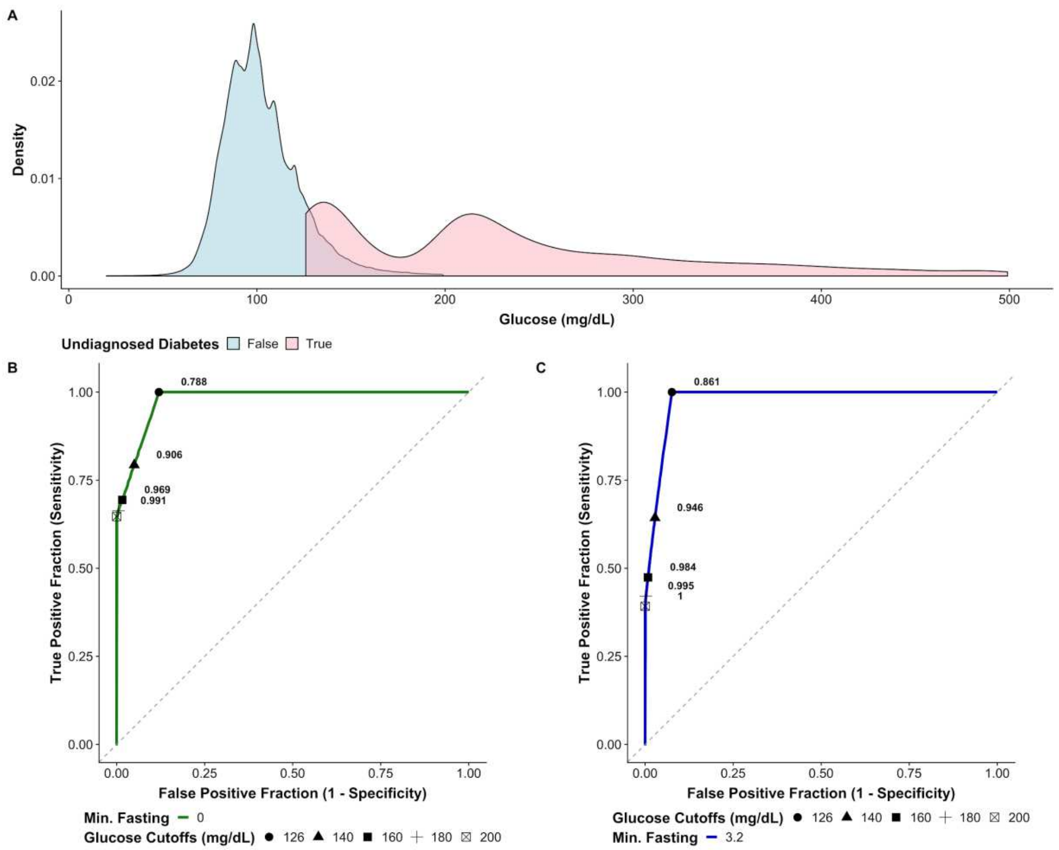

Figure S3. Glucose distributions in undiagnosed diabetes and Receiver Operator Characteristic (ROC) curve showing the ratio of the false positive and the true positives for different glucose cutoffs. (A) shows the glucose distribution for the individuals with no diabetes and those with undiagnosed diabetes populations. The bottom plots show the ROC curve for different glucose cutoffs. The numbers on the graph give the overall accuracy of detection for the 5 specific glucose cutoffs - 126 mg/dL, 140 mg/dL, 160 mg/dL, 180 mg/dL and 200 mg/dL - the current standard for diagnosis using random glucose test. (B) shows the accuracy measured for the different glucose cutoffs for no fasting; (C) shows the accuracies for a fasting time for 3.2 hrs - the median fasting time for the cohort.

	Overall	Women	Men
**Sample Size**	750924	651122[1]	99802
**Mean Age (95% CI), years**	30.25 (30.21, 30.29)	30.25 (30.21 30.29)	30.22 (30.13, 30.31)
**Age Categories (95% CI), %**			
**15-24**	33.87 (33.69, 34.05)	33.84 (33.66, 34.02)	34.07 (33.58, 34.56)
**25-34**	29.85 (29.67, 30.03)	29.87 (29.68, 30.06)	29.71 (29.23, 30.19)
**35-44**	25.14 (24.98, 25.3)	25.15 (24.99, 25.31)	25.1 (24.66, 25.54)
**45-49**	11.14 (11.03, 11.25)	11.14 (11.02, 11.26)	11.12 (10.83, 11.41)
**Wealth Index (95% CI), %**			
**Poorest**	17.3 (16.93, 17.67)	17.66 (17.29, 18.03)	14.95 (14.34, 15.56)
**Poor**	19.62 (19.3, 19.94)	19.69 (19.38, 20)	19.1 (18.46, 19.74)
**Middle**	20.77 (20.47, 21.07)	20.68 (20.39, 20.97)	21.4 (20.79, 22.01)
**Rich**	21.31 (20.96, 21.66)	21.2 (20.85, 21.55)	22.02 (21.32, 22.72)
**Richest**	21 (20.46, 21.54)	20.77 (20.24, 21.3)	22.53 (21.52, 23.54)
**Education (95% CI), %**			
**No Education**	25.66 (25.34, 25.98)	27.75 (27.41, 28.09)	11.92 (11.52, 12.32)
**Primary**	12.49 (12.34, 12.64)	12.56 (12.4, 12.72)	12.07 (11.69, 12.45)
**Secondary**	48.72 (48.44, 49)	47.24 (46.95, 47.53)	58.44 (57.86, 59.02)
**Higher**	13.12 (12.84, 13.4)	12.45 (12.18, 12.72)	17.56 (16.96, 18.16)
**Place of Residence (95% CI), %**			
**Urban**	34.63 (33.72, 35.54)	34.23 (33.34, 35.12)	37.25 (35.67, 38.83)
Rural	65.37 (64.46, 66.28)	65.77 (64.88, 66.66)	62.75 (61.17, 64.33)
**Region of Country (95% CI), %**			
**Central**	24.42 (23.78, 25.06)	24.64 (24, 25.28)	22.95 (21.87, 24.03)
**East**	21.59 (20.85, 22.33)	22.02 (21.28, 22.76)	18.77 (17.58, 19.96)
**North**	12.73 (12.26, 13.2)	12.62 (12.15, 13.09)	13.48 (12.66, 14.3)
**NorthEast**	3.49 (3.31, 3.67)	3.52 (3.34, 3.7)	3.26 (2.97, 3.55)
**South**	22.89 (22.1, 23.68)	22.82 (22.04, 23.6)	23.33 (21.99, 24.67)
**West**	14.88 (14.1, 15.66)	14.37 (13.62, 15.12)	18.2 (16.73, 19.67)
**Lifestyle, (95% CI), %**			
**Smokes**	12 (11.8, 12.2)	6.98 (6.82, 7.14)	44.95 (44.26, 45.64)
**Drinks**	4.98 (4.84, 5.12)	1.26 (1.18, 1.34)	29.45 (28.75, 30.15)
**Mean Body Mass Index**	21.96 (21.93, 21.99)	21.87 (21.84, 21.90)	22.59 (22.52, 22.66)
**Mean Glucose (95% CI), mg/dL**	104.92 (104.76, 105.08)	104.67 (104.51, 104.83)	106.56 (106.17, 106.95)
**Mean Systolic BP (95% CI), mmHg**	115.54 (115.45, 115.63)	114.75 (114.66, 114.84.19)	120.71 (120.53, 120.89)
**Mean Diastolic BP (95% CI), mmHg**	78.13 (78.06, 78.20)	77.95 (77.87, 78.03)	79.26 (79.14, 79.38)
**Access to health care (SE), %**			
Yes	38 (37.57, 38.43)	37 (36.57, 37.43)	45 (44.12, 45.88)
No	62 (61.57, 62.43)	63 (62.57, 63.43)	55 (54.12, 55.88)

Table S1: Survey-adjusted characteristics for the men and women cohort from NFHS-4/DHS India, 2015-2016.

[1] Sample size for non-pregnant women

	Overall (95% CI)	Women (95% CI)	Men (95% CI)
Self Report Diabetes (N=10686)	58 (56.6, 59.3)	59.5 (58.0, 61.0)	49.5 (46.3, 52.8)
Uncontrolled Diabetes (N=1926)	18.6 (17.3, 19.9)	18.4 (17.0, 19.8)	20 (16.7, 23.4)
Undiagnosed Diabetes (N=8192)	42 (40.7, 43.4)	40.5 (39.1, 42.0)	50.5 (47.2, 53.7)
Undiagnosed Diabetes, Random (N=5299)	27.6 (26.5, 28.6)	26.5 (25.3, 27.6)	33.5 (30.7, 36.4)
Undiagnosed Diabetes, Fasting (N=2893)	14.5 (13.6, 15.4)	14 (13.1, 15.0)	16.9 (14.8, 19.1)
Undiagnosed with healthcare access (N=3417)	44.6 (42.8, 46.4)	42.7 (40.8, 44.6)	53 (48.9, 57.1)

Table S2: Survey-adjusted proportions of diabetes for men and women identified as having diabetes in the NFHS-4/DHS India, 2015-2016. Random: random glucose level ≥ 200mg/dL. Fasting: random glucose level ≥ 126mg/dL and time since last ate ≥ 8 hours.

	Undiagnosed Diabetes		
	All	Random	Fasting
**Unweighted Sample Size (Diabetes N = 18878)**	8192	5299(8192)	2893(8192)
Sex, (95% CI), %			
**Female**	82 (80.61, 83.39)	81 (79.31, 82.69)	82 (79.8, 84.2)
Male	18 (16.61, 19.39)	19 (17.31, 20.69)	18 (15.8, 20.2)
**Mean Age (95% CI), years**	37.79 (37.516, 38.064)	39.37 (39.07, 39.67)	34.79 (34.289, 35.30)
**Place of Residence (95% CI), %**			
**Urban**	44 (41.92, 46.08)	46 (43.67, 48.33)	40 (36.57, 43.43)
Rural	56 (53.92, 58.08)	54 (51.67, 56.33)	60 (56.57, 63.43)
**Region of Country (95% CI), %**			
**Central**	19 (17.82, 20.18)	18 (16.69, 19.31)	21 (18.98, 23.02)
**East**	19 (17.26, 20.74)	21 (19.02, 22.98)	16 (13, 19)
**North**	9 (8.2, 9.8)	8 (7.14, 8.86)	11 (9.51, 12.49)
**NorthEast**	2 (1.73, 2.27)	3 (2.61, 3.39)	1 (0.8, 1.2)
**South**	34 (31.98, 36.02)	37 (34.67, 39.33)	29 (25.92, 32.08)
**West**	16 (14.22, 17.78)	13 (11.28, 14.72)	22 (18.69, 25.31)
**Smokes (95% CI), %**	15 (13.78, 16.22)	16 (14.55, 17.45)	14 (12.04, 15.96)
**Drinks (95% CI), %**	8 (7.06, 8.94)	8 (6.9, 9.1)	7 (5.55, 8.45)
**Mean Body Mass Index (95% CI), kg/m^2**	25.25 (25.06, 25.44)	26.18 (25.96, 26.40)	23.47 (23.16, 23.78)
**Mean Glucose (95% CI), mg/dL**	234.01 (230.67, 237.35)	282.71 (279.36, 286.04)	141.39 (140.57, 142.21)
**Mean Systolic BP (95% CI), mmHg**	125.36 (124.78, 125.94)	128.26 (127.55, 128.97)	119.84 (118.97, 120.71)
**Mean Diastolic BP (95% CI), mmHg**	84.87 (84.28, 85.45)	87.11 (86.30, 87.92)	80.62 (79.979, 81.26)
**Access to health care (95% CI), %**			
Yes	45 (43.26, 46.74)	44 (41.92, 46.08)	45 (42.06, 47.94)
No	55 (53.26, 56.74)	56 (53.92, 58.08)	55 (52.06, 57.94)

Table S3: Survey-adjusted characteristics for random undiagnosed diabetes and fasting undiagnosed diabetes. Random is defined as persons having a random glucose level ≥ 200mg/dL. Fasting is defined as persons having random glucose level ≥ 126 mg/dL and not eating (fasting) for ≥ 8 hours.

	Diabetes vs All Prevalence Ratio (95% CI)	Undiagnosed vs Self Report Prevalence Ratio (95% CI)	Undiagnosed with Health Care Access vs Undiagnosed Without Health Care Access Prevalence Ratio (95% CI)
	Univariate	Univariate	Univariate
Sex (Ref: Male)			
Female	0.84 (0.78,0.89)***	0.8 (0.75,0.86)***	0.81 (0.74,0.88)***
Age Category (Ref: 15-24)			
25-34	2.53 (2.31,2.77)***	0.92 (0.84,1.01)	1.38 (1.19,1.61)**
35-44	6.46 (5.92,7.05)***	0.86 (0.79,0.94).	1.4 (1.21,1.61)***
45-49	11.67 (10.62,12.83)***	0.76 (0.69,0.84)***	1.35 (1.17,1.56)*
Wealth (Ref: Poorest)			
Poor	1.24 (1.13,1.35)***	1.06 (0.97,1.16)	1.1 (0.96,1.27)
Middle	1.68 (1.53,1.83)***	1.05 (0.95,1.15)	1.23 (1.08,1.41)
Rich	2.46 (2.25,2.69)***	0.88 (0.79,0.97)	1.27 (1.12,1.45)*
Richest	2.91 (2.66,3.18)***	0.75 (0.68,0.83)***	1.18 (1.02,1.35)
Education Level (Ref: no education)			
Primary	1.16 (1.08,1.24)*	0.86 (0.79,0.92)*	1.04 (0.93,1.16)
Secondary	0.89 (0.84,0.94)**	0.82 (0.77,0.87)***	0.96 (0.89,1.05)
Higher	0.88 (0.8,0.96)	0.8 (0.73,0.89)**	1 (0.87,1.14)
Blood Pressure Cuff Size (Ref: Small)			
Medium	2.36 (2.17,2.57)***	0.91 (0.83,1)	1.17 (1.01,1.36)
Large	6.74 (6.03,7.52)***	0.94 (0.83,1.07)	1.35 (1.11,1.64)
Body Mass Index	1.07 (1.07,1.07)***	1 (0.99,1)	1.01 (1,1.02).
Lifestyle			
Smokes (Ref: Doesn't smoke)	1.2 (1.13,1.28)***	1.09 (1.02,1.17)	1.28 (1.17,1.4)***
Drinks (Ref: Doesn't drink)	1.54 (1.4,1.69)***	1.06 (0.96,1.18)	1.45 (1.32,1.6)***
Residence (Ref: Urban)			
Rural	0.56 (0.53,0.59)***	1.21 (1.13,1.3)***	1.02 (0.94,1.1)
Region (Ref: Central)			
East	1.34 (1.24,1.45)***	0.86 (0.79,0.93).	0.91 (0.8,1.03)
North	1.08 (1.01,1.15)	0.84 (0.78,0.91)**	1.06 (0.95,1.18)
North-East	0.98 (0.9,1.07)	0.85 (0.78,0.93).	0.74 (0.63,0.88).
South	2.51 (2.34,2.68)***	0.76 (0.7,0.82)***	1.52 (1.41,1.65)***
West	1.35 (1.24,1.47)***	1.01 (0.93,1.11)	0.71 (0.59,0.85)*
Health Care	1.49 (1.42,1.57)***	0.89 (0.84,0.94)*	NA
Health Insurance	1.52 (1.44,1.61)***	1.02 (0.96,1.08)	4.56 (4.24,4.9)***

Table S4: Unadjusted prevalence ratios for persons with undiagnosed diabetes. Prevalence ratios for all persons with diabetes is given for comparison. Bonferroni adjusted p.values are denoted as follows: corrected p value < 0.0001 (***), corrected p value < 0.001 (**), corrected p value < 0.01 (*), corrected p value < 0.05 (.). Factors that are associated with increased prevalence of undiagnosed (diabetes, undiagnosed with health care access) are shown in red. Factors associated with decreased prevalence of undiagnosed (diabetes, undiagnosed with health care access) are shown in blue. Factors that are not significant are given in black.

	Diabetes vs All Prevalence Ratio (95% CI)		Undiagnosed vs Self Report Prevalence Ratio (95% CI)		Undiagnosed with Health Care Access vs Undiagnosed without Health Care Access Prevalence Ratio (95% CI)	
	Women	Men	Women	Men	Women	Men
Age (Ref: 15-24)						
25-34	1.94 (1.76,2.15)***	2.21 (1.79,2.71)***	0.88 (0.79,0.97)	1.02 (0.85,1.22)	1.32 (1.12,1.56).	1.2 (0.88,1.62)
35-44	4.31 (3.9,4.75)***	5.76 (4.78,6.94)***	0.81 (0.73,0.9)**	0.93 (0.78,1.1)	1.2 (1.02,1.41)	1.49 (1.13,1.97)
45-49	7.5 (6.71,8.39)***	9.25 (7.57,11.31)***	0.72 (0.64,0.81)***	0.79 (0.65,0.95)	1.19 (1,1.42)	1.14 (0.84,1.56)
Wealth (Ref: Poorest)						
Poor	1.1 (1,1.21)	1.13 (0.92,1.38)	1.08 (0.98,1.2)	1.16 (0.94,1.41)	1.04 (0.9,1.21)	1.01 (0.74,1.37)
Middle	1.23 (1.12,1.36)**	1.45 (1.17,1.78).	1.11 (0.99,1.24)	1.29 (1.06,1.57)	1.13 (0.97,1.32)	1.15 (0.87,1.52)
Rich	1.56 (1.41,1.73)***	1.66 (1.35,2.03)***	0.93 (0.83,1.05)	1.27 (1.03,1.56)	1.17 (1,1.37)	1.15 (0.87,1.53)
Richest	1.68 (1.5,1.89)***	2.17 (1.74,2.72)***	0.79 (0.69,0.91).	1.15 (0.93,1.44)	1.18 (0.98,1.42)	1.12 (0.83,1.51)
Education Level (Ref: no education)						
Primary	1.15 (1.07,1.24)*	1.28 (1.03,1.59)	0.83 (0.76,0.9)**	0.94 (0.78,1.13)	0.99 (0.89,1.11)	1.21 (0.94,1.56)
Secondary	1.03 (0.96,1.09)	1.33 (1.1,1.6)	0.8 (0.74,0.86)***	0.82 (0.7,0.96)	0.94 (0.85,1.04)	1.12 (0.91,1.38)
Higher	0.89 (0.79,0.99)	1.15 (0.92,1.44)	0.89 (0.78,1.01)	0.69 (0.56,0.86).	0.99 (0.82,1.2)	1.26 (0.99,1.61)
Body Mass Index	1.08 (1.08,1.09)***	1.03 (1.03,1.04)***	1.01 (1.01,1.02)**	1.01 (1,1.02)	1 (1,1.01)	1 (0.99,1.01)
Lifestyle						
Smokes (Ref: No Smoking)	1.1 (1,1.21)	0.98 (0.87,1.1)	0.88 (0.79,0.98)	1.03 (0.92,1.16)	1.53 (1.36,1.72)***	1.22 (1.05,1.41)
Drinks (Ref: No Drinking)	1.38 (1.06,1.79)	1.07 (0.96,1.2)	0.83 (0.63,1.09)	0.96 (0.86,1.08)	1.31 (1.11,1.54).	1.11 (0.96,1.29)
Residence (Ref: Urban)						
Rural	0.84 (0.79,0.9)***	1.03 (0.9,1.19)	1.1 (1.02,1.19)	0.98 (0.85,1.13)	1.09 (0.99,1.2)	1.07 (0.92,1.25)
Region (Ref: Central)						
East	1.4 (1.3,1.52)***	1.55 (1.31,1.84)***	0.82 (0.74,0.9)**	0.89 (0.76,1.04)	0.94 (0.82,1.08)	0.87 (0.67,1.13)
North	0.82 (0.77,0.88)***	0.87 (0.74,1.02)	0.89 (0.82,0.97)	0.87 (0.74,1.01)	1.12 (0.99,1.27)	0.99 (0.79,1.24)
North-East	0.95 (0.86,1.04)	1.08 (0.88,1.33)	0.9 (0.81,1.01)	0.81 (0.65,1)	0.61 (0.51,0.74)***	0.92 (0.69,1.23)
South	1.63 (1.5,1.76)***	1.76 (1.51,2.06)***	0.8 (0.73,0.88)***	0.78 (0.66,0.93)	1.59 (1.44,1.74)***	1.4 (1.18,1.67)*
West	1.02 (0.92,1.12)	1.01 (0.84,1.22)	1.07 (0.96,1.19)	1.07 (0.92,1.25)	0.61 (0.49,0.77)**	1.01 (0.79,1.3)
Health Care	1.11 (1.04,1.17).	1.27 (1.14,1.42)**	0.95 (0.89,1.01)	0.82 (0.74,0.91)*	NA	NA

Table S5: Adjusted prevalence ratios for women and men with undiagnosed diabetes. Adjusted prevalence ratios for persons with diabetes is provided as a reference. Bonferroni adjusted p.values are denoted as follows: corrected p value < 0.0001 (***), corrected p value < 0.001 (**), corrected p value < 0.01 (*), corrected p value < 0.05 (.). Factors that are associated with increased prevalence of undiagnosed (diabetes, undiagnosed with health care access) are shown in red. Factors associated with decreased prevalence of undiagnosed (diabetes, undiagnosed with health care access) are shown in blue. Factors that are not significant are given in black.

	Diabetes vs Healthy		Undiagnosed vs Self Report		Undiagnosed with Health Care Access vs Undiagnosed without Health Care Access	
	Women	Men	Women	Men	Women	Men
Glucose (95% CI), mg/dL	90.52 (87.47, 93.56)***	96.746 (90.94, 102.55)***	66.70 (61.60, 71.79)***	61.34 (50.83, 71.85)***	-10.39 (-17.39, -3.39)	-16.70 (-29.35, -4.05)
Systolic BP (95% CI), mmHg	4.31 (3.91, 4.70)***	3.86 (2.93, 4.80)***	2.38 (1.57, 3.19)***	1.96 (0.24, 3.66)	-0.53 (-1.77, 0.71)	-0.93 (-3.02, 1.16)
Diastolic BP (95% CI), mmHg	2.53 (2.11, 2.94)***	2.69 (2.05, 3.33)***	1.38 (0.50, 2.26)	1.23 (0.054, 2.41)	-0.36 (-1.60, 0.89)	-0.73 (-2.13, 0.67)
Self Report:						
Hypertension (95% CI), %	7.7 (6.7, 8.7)***	6.0 (4.1, 8.0)***	-4.4 (-6.4, -2.4)*	-2.9 (-6.9, 10.0)	1.4 (-1.4, 4.2)	0.1 (-5.1, 5.2)
Heart Disease (95% CI), %	4.1 (3.4, 4.8)***	6.7 (4.11, 9.4)**	-8.0 (-9.2, -6.8)***	-13.7 (-18.1, -9.2)***	1.3 (0.2, 2.3)	1.7 (1, 3.2)
Thyroid Disorder (95% CI), %	4.0 (3.1, 4.8)***	6.3 (3.7,9.)*	-6.7 (-8.2, -5.2)***	-11.7 (-16.2, -7.1)**	3.4 (1.5, 5.2)	1.2 (-1.2, 3.6)

Table S6: Comparison of the standardized mean differences in key measurements and co-morbidities between the undiagnosed and self report groups. Comparison of the diabetes and the healthy population is provided as reference. Bonferroni corrected p.values are denoted as follows: corrected p value < 0.0001 (***), corrected p value < 0.001 (**), corrected p value < 0.01 (*), corrected p value < 0.05 (.). Factors that are associated with increased prevalence of undiagnosed (diabetes, undiagnosed with health care access) are shown in red. Factors associated with decreased prevalence of undiagnosed (diabetes, undiagnosed with health care access) are shown in blue. Factors that are not significant are given in black.
